# Enteric bacteria, methicillin resistant *S. aureus* and antimicrobial susceptibility patterns from buses surfaces in Mekelle city, Tigray, Ethiopia

**DOI:** 10.1186/s13104-019-4366-1

**Published:** 2019-06-13

**Authors:** Atsebaha Gebrekidan Kahsay, Solomon Weldegebreal Asgedom, Haftom Legesse Weldetinsaa

**Affiliations:** 1 Department of Medical Microbiology and Immunology, School of Medicine, College of Health Sciences, Mekelle University, Mekelle, Ethiopia; 2 School of Pharmacy, College of Health Sciences, Mekelle University, Mekelle, Ethiopia; 3 Department of Medical Laboratory Sciences, Adigrat University, Adigrat, Ethiopia

**Keywords:** Enteric bacterial, *Escherichia coli*, *Staphylococcus aureus*, Antimicrobial susceptibility test, Mekelle, Bus

## Abstract

**Objective:**

To assess the enteric bacteria, methicillin resistant *S. aureus* and antimicrobial susceptibility patterns from buses surfaces in Mekelle, Tigray, Ethiopia.

**Results:**

A total of 300 swab samples were collected from the handle surfaces of the six city buses. The bacterial isolates revealed from the swab samples were *E. coli*, *Enterobacter* spp. and *S. aureus.* The overall positivity rates of *E. coli*, *Enterobacter* spp. and *S. aureus* were 8 (4%), 4 (1.3%) and 54 (18%) respectively. Methicillin resistant *S. aureus* was seen in 17 (5.7%) of the total 300 swab samples collected and 17 (31.5%) of the *S. aureus* isolates. All (100%) of the isolates of *E. coli* and *Enterobacter* spp. showed resistance for ampicillin and three-fourth of the isolates of *E. coli* and *Enterobacter* spp. displayed resistance for chloramphenicol (75%). Five antimicrobials (ampicillin, chloramphenicol, tetracycline, ciprofloxacin, and cotrimoxazole) have showed resistant for one isolate of *E. coli.* Likewise four antimicrobials (ampicillin, chloramphenicol, ciprofloxacin, and cotrimoxazole) have revealed resistant for one isolate of *Enterobacter* spp. Moreover, three isolates of *S. aureus* were also found resistance to four antibiotics.

## Introduction

Transportation network systems are important to transport passengers from one area to the other area. With the expansion of transportation network systems, parallel expansions of communicable diseases in the globe are fashionable from time to time [1]. These transportation networks leave people at risk from the emergence of new strains of diseases in the globe [2]. This would be the worst if the pathogenic microorganisms developed drug resistance. Many studies were conducted all over the world to isolate pathogenic microorganisms from the different surface like public hand touch surfaces of the bus, train, mobile phone, hand knob, hospital, shopping cart [3, 4]. Some of the common pathogenic bacteria isolated from hand touch surfaces are *E. coli, Salmonella* and multi-drug resistant *S. aureus* [5–7].

*Escherichia coli* are predominately isolated from different surfaces [8] and hand touch surface of public buses. *Salmonella typhi* and *Shigella* species are the second and third isolates from hand touch surface of public buses [9]. The transmission of *S. aureus* is facilitating during crowdedness, significant hand to surface contact and failure to wash hands immediately after dropping from the bus [10]. The infection due to *S. aureus* varies from a simple skin infection to the serious and fatal bacteremia and pneumonia [11]. The contribution of *S. aureus* infection in the surgical site and ventilator-associated pneumonias are 30% and 24% respectively [12]. The serious problem of *S. aureus* is its emergence of drug resistance [13].

This study tried to assess different search engines to find similar published researches in Ethiopia and Africa but unable to find such a research and not included the previous overview of enteric bacteria and MRSA in the study area. But, due to the fast growing of the Mekelle city, many young individuals are flowing from the rural area to the city and the city administration is introducing city buses to minimize the shortage of transportation. In line with the transportation, we conducted a research to assess the pathogenic enteric bacteria and MRSA from the handle surfaces of the city buses in the Mekelle city.

## Main text

### Materials and methods

Mekelle, the capital city of Tigray region, is located 784 km north of Addis Ababa. It is a major economic and educational centre, with a new international airport, federal university teaching hospital and it is the home for Mekelle University. It is also the centre of industrial parks. The city has a huge natural, historic and religious sites and its population growth is increasing from time to time and has an estimated population of 286,600 [14]. It is also an entry point to the Gere Alta landscape and rock-hewn churches and the breathtaking Dallol area in the Afar Region. Due to an increase in the population size in the city, the transportation service becomes an urgent demand.

#### Study design and period

The cross-sectional study design was carried out among the city buses surfaces in January and February 2017.

#### Study subjects and sampling technique

Six city buses were included in the study. These buses have selected purposively since they are able to transport long distance in the city and able to transport about 100 individuals at a time. Swab samples were taken while the bus completed one trip (at the end of the travel). A single bus has a total of 100 handle surfaces (sit and stand). Swabbing of the handle surfaces was started from the front door by leaving one sit and taking another sit and finally to have 50 swab samples per the single bus. The way of sample collection for all the buses included in this study was similar. Of the total 600 handle surfaces in all the selected city buses, swab samples were collected from the 300 handle surfaces.

#### Swab sample collection

A 5 ml sized test tube having 2 ml peptone water with application cotton swab was sterilized at 121 °C with 15 mmHg for 15 min in the autoclave [15]. The prepared peptone water broth was put in the ice box while we were going to the swab sample collecting site. The buses were given code numbers from one to six. We designed our protocol to collect 10 swab samples per day excluding Saturday and Sunday. As per the sampling technique, we have recruited 50 samples from Monday to Friday from the bus given the first code number and continued in a similar way to employ a total of 300 swab samples. While we were labeling and collecting the swab samples, we started from the front handle surface near the driver. Once we collected 10 swab samples aseptically from the handle surface, we put in the icebox and transported them into Microbiology laboratory in Mekelle University College of Health Sciences, Department of Medical Microbiology and Immunology.

#### Isolation and identification of bacteria

Once arrived in the Microbiology laboratory, the swab samples were inoculated directly into MacConkey agar and Mannitol salt agar and incubated at 37 °C for 24 h.

After overnight incubation, colonies grown on MacConkey agar were identified and characterized using a series of biochemical tests including oxidase test, urease test, Killer Iron Agar, Indole, Citrate, and also motility test. Although this study hypothesized to assess the enteric bacteria including *E. coli* (may indicate fecal contamination), *Shigella* and *Salmonella* but *Shigella* and *Salmonella* were not isolated from MacConkey agar. Unfortunately *E. coli and Enterobacter* spp. were the only bacteria recovered from the swab samples collected.

On the other hand, colonies grown on Mannitol salt agar and having golden yellowish color were identified using gram stain and characterized by biochemical tests including catalase and coagulase tests. Gram-positive in cluster form, catalase positive and coagulase positive colonies were confirmed as *S. aureus*. Cefoxitin disk was used to identify methicillin-resistant *S. aureus* [16].

#### Antimicrobial susceptibility tests

The isolated bacteria were subjected to the antimicrobial susceptibility tests using Kirby Bauer disk diffusion method. Mueller–Hinton agar (HI Media Laboratories, Pvt. Mumbai, India) was used to determine the susceptibility testing. All the antimicrobial disks used were imported from Oxide Ltd. Basingstoke Hampshire England.

The antimicrobial disks used to test bacterial isolates in this study were ampicillin, penicillin, erythromycin, chloramphenicol, ciprofloxacin and cefoxitin, tetracycline and cotrimoxazole. Results were reported as sensitive, intermediate and resistance following clinical laboratory standards institute [16].

#### Quality control

During sample collection, we used candle jar to prevent other microorganisms from joining from the air. Each lot and shipment of a medium was checked for expiry date prior to use as part of quality control. The only medium which has passed quality control was used for testing. The performance of the media and antimicrobial disks were checked by *E. coli* strain American Type Culture Collection 25922 (ATCC 25922) and *S. aureus* (ATCC-25923) [16].

#### Data analysis

Data were entered and analyzed using SPSS version 20. Descriptive statistics were calculated into frequencies and percentages and summarized in the table.

#### Ethical consideration

Ethical clearance was obtained from the Ethical Review Committee of Mekelle University, College of Health Sciences. Permission was collected from Mekelle city administrative transport office.

### Results

#### Bacterial isolates

A total of 300 swab samples were collected from the handle surfaces of the six city buses. The bacterial isolates revealed from the swab samples were *E. coli*, *Enterobacter* spp. and *S. aureus.* The overall positivity rates of *E. coli*, *Enterobacter* spp. and *S. aureus* were 8 (4%), 4 (1.3%) and 54 (18%) respectively. A cumulative of 66 (22%) bacteria was recovered from the total of 300 swab samples. Methicillin resistant *S. aureus* was seen in 17 (5.7% %) of the total 300 swab samples collected and 17 (31.5%) of the *S. aureus* isolates.

#### Antimicrobial susceptibility testing

Bacterial isolates were tested for antimicrobial drugs. These were ampicillin, erythromycin, chloramphenicol, ciprofloxacin, cefoxitin, cotrimoxazole and tetracycline. The resistance patterns of *S. aureus*, *E. coli* and *Enterobacter* spp. isolates were explained in Table 1.

**Table 1:** Antimicrobial Resistance patterns of *S. aureus, E. coli* and *Enterobacter* spp. among city buses surfaces in Mekelle city, Tigray, Ethiopia, 2017

Antimicrobial agent	*S. aureus,* N = 54		*E. coli,* N = 8		*Enterobacter* spp., N = 4	
	R	S	R	S	R	S
	No (%)	No (%)	No (%)	No (%)	No (%)	No (%)
Penicillin	40 (74.1)	14 (25.9)	–	–	–	–
Ampicillin	–	–	8 (100)	0 (0)	4 (100)	0 (0)
Erythromycin	6 (11.1)	48 (88.9)	0 (0)	8 (100)	0 (0)	4 (100)
Chloramphenicol	37 (68.5)	17 (31.5)	6 (75)	2 (25)	3 (75)	1 (25)
Ciprofloxacin	6 (11.1)	48 (88.9)	3 (37.5)	5 (62.5)	1 (25)	3 (75)
Cefoxitin	17 (31.5)	37 (68.5)	–	–	–	–
Cotrimoxazole	13 (24.1)	41 (75.9)	1 (12.5)	7 (87.5)	2 (50)	2 (50)
Tetracycline	3 (5.6)	51 (95.4)	6 (75)	2 (25)	0 (0)	4 (100)

*R* resistant, *S* susceptible

A single isolate of *E. coli* showed resistance to multiple antimicrobial agents (ampicillin, chloramphenicol, ciprofloxacin, cotrimoxazole and tetracycline) and in a similar way one isolate of *Enterobacter* spp. displayed resistance to four (ampicillin, chloramphenicol, cotrimoxazole, ciprofloxacin) antimicrobial drugs (Table 2). Three isolates of *S. aureus* were resistant to four antibiotics “Table 3“.

**Table 2:** Multidrug resistant patterns of *E. coli and Enterobacter* spp. among city buses surfaces in Mekelle city, Tigray, Ethiopia, 2017

Antimicrobial agents	Resistant to *E. coli*	Isolates	Resistant to *Enterobacter* spp.	Isolates
Three	AMP, CAF, TT	3	AMP, CAF, COT	2
Four	AMP, CAF, TT, CIP	3	AMP, CAF, COT, CIP	1
Five	AMP, CAF, TT, CIP, COT	1		

*AMP* ampicillin, *CAF* chloramphenicol, *COT* cotrimoxazole, *TT* tetracycline, *CIP* ciprofloxacin

**Table 3:** Multidrug resistant patterns of *S. aureus* among city buses surfaces in Mekelle city, Tigray, Ethiopia, 2017

Number of antimicrobial resistance	*S. aureus*	
	Resistance antibiogram	Number of isolates
Three	P, CAF, COT	1
	P, ERY, CIP	2
	ERY, CAF, CX	2
	CAF, CX, TT	1
	CAF, CX, COT	1
Four	P, ERY, CAF, CIP	1
	P, CAF, COT, TT	1
	CAF, CX, COT, TT	1

*P* penicillin, *ERY* erythromycin, *CAF* chloramphenicol *CEX* cefoxitin, *COT* cotrimoxazole, *TT* tetracycline, *CIP* ciprofloxacin, *Cx* cefoxitin

### Discussion

With the expansion of transportation network systems, parallel expansions of communicable diseases in the globe are happening from time to time. These transportation networks leave people at risk from the emergence of new strains of diseases in the globe. Some of the common pathogenic bacteria isolated from hand touch surfaces in different studies are *E. coli, Salmonella* and multi-drug resistant *S. aureus* [5–7]. These are medically important bacteria that anyone can acquire whenever there is poor personal hygiene and this is very common in low-income countries and leads to diarrhoea [17, 18]. The problem is not only their acquisition as causative agents of diarrhea but also their lack of sufficient treatment due to the emergence of drug resistance [19, 20]. Although this study is unable to identify the most common enteric bacterial pathogens (*Shigella* and *Salmonella*), *E. coli* is an indication that there might be faecal contamination of bus surfaces in the study area. This can be a good opportunity for the spreading of enteric pathogens in the study area. On the other hand, even if it is clear that *S. aureus* is a skin and a nasal flora, its magnitude of infection is from a simple skin infection to a serious infection like pneumonia and septicemia [11, 12]. Moreover, another serious problem of *S. aureus* is its emergence of drug resistance (the emergence of methicillin resistance *S. aureus*) [13]. By its nature, MRSA carrier people may have contact with their nasal secretion by their hand and automatically handle the surface of the bus and as soon as possible another passenger comes and handle the same handle surfaces. This might be a good network for the transmission of MRSA from one to the other.

In the present study, three genuses of bacteria were isolated. These are *E.* coli, *Enterobacter* spp. and *S. aureus*. Similar to the present study *E. coli* and *S. aureus* were isolated in Bangladesh however unlike to this study *Shigella* and *Salmonella* was revealed in Bangladesh [9].

Two-third of the isolates of *S. aureus* were resistance to MRSA. This result is in line with a study conducted in Portugal [10]. Above 18% of *S. aureus* isolates seems to appear resistant to three and four antimicrobial agents that implies multidrug resistance. One isolate of *E. coli* and *Enterobacter* spp. showed resistance to five and four antimicrobial drugs respectively. Moreover, three isolates of *S. aureus* were also found resistance to four antibiotics.

### Conclusion

Although the common enteric bacterial pathogens (*Shigella* and *Salmonella*) were not revealed from the hand touch surfaces of city buses, the recovery of *E. coli* may indicate the faecal contaminations along the bus surfaces. Multidrug-resistant isolates of *E. coli* were recovered. This study recommends to passengers that they should wash hands before eating to prevent contamination by the enteric bacteria which may induce gastrointestinal diseases. About 5.6% of the total swab samples and 31.5% of the *S. aureus* isolates were resistant to methicillin.

## Limitations

This study lacks the analysis of mecA gene of *S. aureus* which was mandatory to confirm the cefoxitin disc diffusion method.

## Data Availability

Not applicable.

## Abbreviations

SPSS: statistical software for social sciences
MRSA: methicillin resistance *S. aureus*
CLSI: Clinical Laboratory Standards Institutes
*E. coli*: *Escherichia coli*
*S. aureus*: *Staphylococcus aureus*
